# Community members’ experiences training as medical journal reviewers

**DOI:** 10.1186/s40900-023-00482-x

**Published:** 2023-08-14

**Authors:** Cyleste C. Collins, Erika Hood, Jeri Jewett-Tennant, Kurt Stange, Ashwini R. Sehgal

**Affiliations:** 1 School of Social Work, Cleveland State University, Cleveland, USA; 2 See You at the Top, Cleveland, USA; 3 Case Center for Reducing Health Disparities, Case Western Reserve University, Cleveland, USA; 4 Case Western Reserve University, Cleveland, USA

**Keywords:** Community engaged research, Medical research, Case study, Research dissemination, Patient and public involvement

## Abstract

**Purpose:**

Although medical research dissemination is intended to benefit members of society, few members of society actually participate in the process of publishing findings. This study shares findings from community members’ (including patients and the public) experiences being trained as medical journal reviewers.

**Methods:**

We analyzed findings from two focus group interviews of community reviewers (N = 29) to identify themes in their experiences with the training program.

**Results:**

Community members trained as journal reviewers appreciated learning the context under which manuscript development and review occur from authors and funders, the value of the community member perspectives to science, and strengthened their critical thinking skills. A range of training tools and strategies included glossaries of research terms, creating review guides, practicing reviews, being trained by a supportive team, and working with and learning collaboratively.

**Conclusions:**

Training as a journal reviewer has a positive impact on participating community members. Programs training community members as journal reviewers should incorporate guest speakers well-versed in community engaged research, group activities, a variety of training tools and materials, and highly supportive training teams.

## Abstract (plain language summary)

The findings of medical research are supposed to benefit society, but few members of society that are not specialists actually help publish findings. In this study we hoped to learn about the experiences of community members who were trained to be medical journal reviewers. We interviewed 29 of the 34 community members who were trained to learn what they liked and did not like about the training. We learned that community members appreciated learning about how journal articles get published. Learning from journal article authors was helpful, as well as from people who work for organizations that finance the research. They also learned about how patient and community perspectives are important in science. They also said they learned to strengthen their critical thinking skills. They mentioned training tools and strategies that would have helped them. These included lists of research terms and their definitions, review guides, practicing reviews ahead of time, having supportive trainers, and working with and learning from each other. We conclude that training as a journal reviewer has had a positive impact on the community members. We suggest that programs who want to train community members as journal reviewers should incorporate guest speakers who understand community engaged research. They should also make sure they incorporate group activities, a variety of training tools and materials, and be very supportive.

## Introduction

Clinicians or researchers typically review manuscripts submitted to medical journals, without input from patients or other community members. However, U.S.-based research on how patients and the public (here referred to as “community members”) contribute to other research phases suggests they provide a distinct perspective on research and contribute expertise on their communities [1, 2]. For example, community-based participatory research (CBPR) involves communities in selecting research topics, collecting data, and interpreting findings [3, 4]. Integrating community perspectives into research can lead to a stronger understanding of community priorities, adjusting study designs to minimize participant burden and increase participant recruitment and retention [2, 5–10]. Because journal articles are seen as primarily academic, community members do not generally serve as journal manuscript reviewers. However, community members’ unique perspectives inform and broaden research dissemination activities’ scope, offering journal authors a unique point of view they might not otherwise consider [3, 11].

Physicians, researchers, or other scientific professionals are the primary editors and reviewers of medical journal manuscripts; community members are rarely involved in reviews. Communities tend to be involved with research in early research phases (e.g., selecting research questions, developing study protocols), less common in later phases (e.g., disseminating and implementing findings), and rare in medical journal manuscript reviews [12]. We are aware of only two medical journals (both based in the United Kingdom) that include patients/community members as part of the review process (*British Medical Journal* and *Research Involvement and Engagement*) [13, 14]. We are not aware of efforts in other countries besides the U.S. to include patients/community members in other parts of the scientific process. Some perceive that community members simply do not know enough about research to meaningfully contribute to the research enterprise, including lacking objective viewpoints and statistical training [3]. However, relying solely on scientific reviewers overlooks critical community perspectives [15, 16].

The NIH-funded (U54 mechanism) randomized controlled trial within which this study was embedded found that community members could be successfully trained to meaningfully contribute to disseminating research through their journal manuscript reviews, as compared to using only scientific reviewers [17]. This study describes the experiences the community members who participated in that study had in being trained to review medical journal manuscripts. The study is intended to determine best practices for community training that could be adapted to other CBPR efforts and helps establish training protocols for future community reviewing in other journals.

## Methods

### Project description

Community reviewers participated in-person in 90-min training sessions per week for 6 weeks in spring 2018 and fall 2019. The training topics are listed in Table 1. The training included didactic teaching, and interactive and skill-building exercises to enhance writing and critical thinking skills. Trainees reflected on relevant personal experiences to help them identify values, beliefs, and biases they might bring to their reviews [18, 19]. The trainees practiced by reviewing manuscripts which the project team evaluated and suggested areas to improve. Trainees received $50 for each training session they attended.

**Table 1:** Community reviewers’ training topics

*Training topics*	
How research questions are formulated	
Study inclusion and exclusion criteria	
Subject recruitment and retention	
Human subjects protection	
Data collection methodologies	
Data analysis overview	
Manuscript sections	
Understanding tables and figures	
Funding and conflicts of interest	
Reviewers’ and editors’ roles	
How to write effective reviews	

### Design

The design was a qualitative case study using a social constructionist inquiry framework to learn about participants’ training experiences [20]. This framework was chosen because we were interested in learning about the participants’ experiences in their own words, as well as the meaning and value of the training.

### Sample and data collection

A total of 34 trainees (Cohort 1, n = 24; Cohort 2, n = 10) including patients, community residents, and community organizations’ staff participated in the training. We recruited new reviewers as previous reviewers left the project which resulted in different cohort sizes. Community members living near Cleveland, Ohio, were recruited for the study through flyers posted at community centers and libraries. Eligibility criteria included: being at least 18 years old, having at least a high school education, having computer access, proficiency in reading and writing in English, demonstrating reading proficiency, and having experience with a common medical condition (e.g., stroke, hypertension, diabetes, etc.) as either a patient or caregiver. Please see Huml et al. [17] for additional details.

#### Survey

A brief 3-question paper-and-pencil survey was created for this study and administered to all 34 participants at the beginning of the training. Questions asked participants to share reasons for participating in the training and their confidence in research topics. The first question was intended to gauge the trainees’ confidence in different research topics (reading research articles; determining the research question, understanding the methods used, understanding tables and figures; identifying potential problems with the research) with “Not at all Confident”; “Somewhat Confident”, “Moderately Confident”, “Very Confident” and “Completely Confident” as response options. The second question was intended to better understand how important they rated reasons for interest in reviewing research articles (learning how to write more effectively; helping authors improve their work; wanting to know what is new in the field of research). Response options included “Not at all Important”; “Slightly Important”; “Moderately Important”; “Very Important”; and “Extremely Important”. The third question was open-ended, asking participants to list any other reasons they were interested in reviewing research articles.

#### Focus group

A total of 29 of the 34 trainees (three men, 26 women) participated in one of two semi-structured focus groups, held in May 2018 (Cohort 1) and October 2019 (Cohort 2). Participants were told about the focus group ahead of time and it was held during each cohort’s last training session. The sample included all trainees who were present on the last day of the cohort’s training. While trainees were free to leave before the focus group began, to our knowledge, none did so. The seven focus group questions asked trainees to describe their experiences with the training, what they learned, what parts of the training were most memorable and helpful, how their experiences in the training had affected them, what they would tell others interested in taking part in the training, and how the training could improve. The Cohort 1 focus group was intended to gather feedback for improving the training for Cohort 2 as well as learning about the trainees’ perspectives on the training.

### Procedures

An institutional review board approved all study activities. An external, Ph.D. level social work researcher unknown to the participants with extensive experience in qualitative and community research facilitated the focus groups; no other project staff were present. The focus groups were held in the trainees’ normal meeting room. The focus groups ranged from 37 to 50 min, were recorded using an MP3 recorder, and a professional transcriptionist transcribed them. All present on the days of the focus groups participated, and none declined. No compensation was offered for participating in the focus group; trainees who attended the session were paid for attending regardless of whether they participated in the focus group.

### Analysis

The outside evaluator conducted thematic analyses of the focus group interviews [21]. The focus group transcripts were examined iteratively and inductively using Atlas ti. Consistent with the social constructionist inquiry framework, we stayed as close to the direct quotes as possible as the analysis unfolded, seeking to locate similarities in training experiences. First, the evaluator created a summary document and shared it with the training team (four individuals) for two reasons: (1) to check the single evaluator/analyst’s interpretations, and (2) to quickly inform the team about improvements needed for future trainings. Next, direct quotes were identified as they related to the research question. The quotes were then examined for common patterns, grouped into categories, and the categories were combined and developed into themes. The two cohorts of data served as source triangulation, as we reviewed each cohort’s data separately, compared the findings and then combined them when we determined the patterns of responses were similar. We established trustworthiness through negative case analysis (in which the data are examined for evidence of inconsistency with larger themes), and the larger project team (five individuals; four directly involved in the training, and one who was external to it) reviewing the findings and looking for inaccuracies, evidence of bias, and/or inconsistencies [20].

## Results

### Survey findings

A total of 34 participants completed the survey (see Tables 2, 3). Most trainees (more than three-quarters) reported participating because they wanted to know what is new in research or to help authors improve their work. More than three-quarters reported they felt confident reading research articles and determining research questions. Half reported they wanted to learn about writing more effectively, and more than half, very or completely confident identifying potential problems with research, and understanding tables, figures, and research methods. In open-ended questions, the trainees wrote that the training was a chance to “be more in tune with medical research,” to “help article authors see things from a different perspective from their own,” and “to provide community voice in academic research.” One said they hoped their input would help reduce health disparities, and another: “I want authors of research articles to be more inclusive in their studies. Medical research …should benefit as many people as possible.” Overall, trainees saw the training as an educational opportunity that could have societal benefits.

**Table 2:** Initial questionnaire: confidence regarding research and motivation for participating in training (N = 34)

	Not at all confident (%)	Somewhat or moderately confident (%)	Very or completely confident (%)
Reading research articles	0	23	77
Determining the research question	0	18	82
Understanding the methods used	3	41	56
Understanding tables and figures	0	41	59
Identifying potential problems with research	0	38	62

**Table 3:** Motivations for participating in training (N = 34)

	Not at all/slightly important (%)	Moderately important (%)	Very important (%)	Extremely important (%)
I want to learn about how to write more effectively	3	38	29	21
I want to help authors improve their work	0	15	41	44
I want to know what is new in the field of research	0	15	44	41

### Qualitative findings: lessons for training community reviewers

A total of 144 codes were generated from which we derived three key lessons learned from the project: context, learning and time, and training tools.

#### Lesson 1: Provide context in which reviews occur

The first lesson we learned was that it is important to provide trainees with the context in which reviews take place. Context was provided through accentuating how community members can contribute to academic publishing and including authors’ and funders’ perspectives in the training. Trainees said they learned how valuable their experiences, expertise, voices, and perspectives are to science. One said: “I like the importance of us wearing our different hats and bringing our own perspective into the research.” Another trainee said, “I never realized it that getting the community involved in [research] from behind the scenes were so important, because you know they asked us that when we review the manuscripts, we should review them from a community perspective.” Better understanding reviews’ contexts, trainees had a new perspective on reading articles.

I never ever read them [before] with like what can be done or ‘What can I say to make this more relevant for the community?’ or to clarify the information. So in that sense, I feel empowered, …I actually have a say in what’s gonna turn out to be the final product. So maybe when I pick up that journal again, it’ll be more tailored and user-friendly.

Bringing in guest speakers, specifically authors and funders, was also helpful for contextualizing the research, manuscript submission, review, and editing process. One guest speaker, an author who shared her experiences in having articles rejected, was seen as “courageous” and “insightful,” and helped trainees better understand how community perspectives contribute to research. “Hearing from an author that they know there’s going to be comments… They’re expecting feedback. They’re expecting it to be not done” was useful. Other guests included research funders who value community-engaged research [e.g., the Patient-Centered Outcomes Research Institute (PCORI)] allowed trainees to more holistically learn about community-engaged research. “That opened up even more doors to us.” The PCORI guest speaker was highlighted as making them feel comfortable through her humility and respect for their perspectives as community members.

#### Lesson 2: Emphasize opportunities for learning and use time carefully

Trainees felt they had learned a great deal from the training, including learning to read with a more critical eye and developing a greater awareness about health disparities. One said they felt the training, “made me a better community member, … I’m just very sensitive to what people are doing and the different populations that are being served and underserved…I just find this extremely valuable.”

Trainees said they now read articles more critically, looking for strengths and weaknesses, and can identify what research leaves out. One trainee said the skills differed from what they do in their daily lives. “You’re really looking at it with a very critical eye and asking a lot of questions, which typically isn’t asked of you in either everyday work or school.”

The trainees noted learning how to review required a learning curve. They said spent substantial time working to understand the highly scientific and technical styles in which articles are written. One said, “the subject matter is not for a layperson… it …took three reads for me.” Others said they had to look up definitions or “had to decipher each word and then put the paragraphs back together again, ‘cause I’m not used to…the way this language is put together.” Because of the effort required, trainees most often suggested accurately informing potential trainees about the time required to conduct reviews and using training time carefully.

Let people know that this isn’t just a “Sit and read it. Sit down and write what you think, or where you’re coming from.” …I mean even if [you’re reviewing using] your frame of reference, you still have to do some research in order to have an intelligent response.

Trainees said the time they spent on homework beyond the in-person training time was more than expected (2–4 h per week), and the turnaround time on homework was also fast. They felt overall, the project demanded a “bigger commitment” than planned. The trainees also noted some in-person training sessions ended early, and they felt the remaining time could have been better utilized by employing training strategies such as group work.

#### Lesson 3: Utilize a variety of training tools

Trainees emphasized how important the various tools used were to their experiences. These tools included the training team, guidelines, glossaries, and other materials provided. They recommended adding group interaction as an additional training tool.

The facilitators were considered key tools. Described as clear, knowledgeable, prepared, organized, patient, helpful, and kind, trainees felt their trainers “totally respected” their questions, even when the same question was asked repeatedly. “There was not one time that anybody was put down… we were asking questions that could be really obvious from their point of view, …they were very, very gracious.” Trainees said trainers responded to their needs and tailored the training to their knowledge base. **“**The facilitators, I thought, were wonderful. They were very patient with us. They made sure you understood. They asked us great questions.”

Trainees said that although the trainers were very helpful, the training’s expectations could have been clearer and training sessions more focused on how to dissect articles. The first cohort suggested providing a research terms glossary to quickly look up unfamiliar terms and reduce time spent researching phrases. “I went looking up the different types of research. …I need a glossary because this is extra.” Based on this feedback, the second cohort received a glossary including basic and complex statistical concepts which they reported was very helpful. Other useful tools included teaching strategies for critical reading, reading for specific keywords, and following a “bullet sheet” guiding trainees through their reviews. Trainees suggested future training should provide more information on how to read and interpret tables and charts. They also suggested getting some information on how to review before their first review to help them feel prepared, “instead of do a review and then learn afterwards what was expected.”

The trainees suggested maximizing group and collaborative opportunities for learning, suggesting that interacting more with fellow trainees would have helped them to learn others’ perspectives in conducting reviews. They suggested strategies and activities such as peer-to-peer conversations to discuss reviews, arranging tables and chairs so they could more easily interact with and help one another, and conducting a group review. Some suggested reviewing the previous cohort’s reviews and discussing them would have helped them begin to think about review structures and approaches. One trainee said that speaking informally with another trainee about a study they both read helped broaden their perspectives on the study.

The most important part of this training came outside of this room when I was talking to another participant …we had come up with different ways of looking at it, and it broadened my perspective. You know I was looking at it one way. …I realized that next time I see something, I have to look at it one way, then I’m gonna have to close my mind, walk away for a while, and then come back and say, “No. Now I have to look at it from a different perspective.”

In one session, the trainees interacted, reviewing one another’s reviews which helped them feel a sense of community with each other and increased their confidence in their reviews.

Finally, trainees highlighted the usefulness of an “open session” held late in the training, when trainees felt more prepared to articulate their questions. The trainers asked the trainees what they still wanted or needed to know before they commenced their independent reviews, allowing trainees to get clarification on remaining questions and see that other trainees had similar questions.

## Discussion

Overall, community journal reviewer trainees viewed their training as extremely useful and valuable. Although trainees expressed some confidence in some aspects of research before the training, they also said they gained skills, including becoming better critical readers of research, and increasing sensitivity to issues facing underserved populations. Trainees benefited from learning from one another, and from people engaged in the writing and research process. Maximizing the effectiveness of training community members as journal reviewers requires contextualizing the review process, especially community contribution, an emphasis on learning, appropriately communicating about and using trainees’ time, and a prepared, knowledgeable, helpful, sensitive, respectful, and responsive training team. Future training should provide trainees with clear expectations of the time required, provide a variety of tools and guidelines for conducting reviews, reading and interpreting tables and charts, and engage trainees in group work.

Including community perspectives in peer-reviewed academic research journal articles is a new and underused practice. Although not currently utilized even in articles that actively include or apply to community members, we found community members are motivated to contribute to creating knowledge through research dissemination. Although they recognized the limits of their research knowledge (specifically with complex statistics) community members are eager to learn and offer useful feedback for applying academic knowledge in community practice. Our findings highlight important process changes to make future community reviewer training programs more effective.

Framing research with community perspectives in mind can broaden the impact, reach, and practical application of research findings. Based on previous research, we assumed community members had unique perspectives reflecting their cultural backgrounds, interpretations of illness, and experiences with specific diseases, perspectives that differ from scientists’ in terms including what research should be prioritized, methods used, and how results should be interpreted [3, 4, 22–26]. Consistent with previous literature, we found community reviewers contributed specific viewpoints on the research beyond the technical aspects. In the training, they indicated learning to value and appreciate their unique contributions while becoming more sensitive to, and engaged with, health disparities in their communities and beyond.

Our findings support the idea that community members can gain basic knowledge about research issues and procedures, an understanding of scientific language and statistical analyses, and can lead to their contributing unique and valuable insight to the existing literature. We found no evidence that our trainees had unrealistic expectations of medical research, nor lacked objectivity [3], but consistent with previous work, they valued research most relevant to them and/or their communities [27]. Utilizing community members’ perspectives can be useful because “researchers ‘don’t know what they don’t know’ until they involve patients/the public.” [28] Furthermore, training community members specifically equips them to be part of the research more actively, as compared with simply engaging with them or including them, symbolically, as a research partner or a member of an editorial board.

### Limitations

One limitation is the potential breach of confidentiality in focus groups; however, our trainees simply offered their perspectives and experiences; no questions were particularly sensitive [29]. The focus group structure allowed the trainees to build on each other’s answers, agreeing and/or offering contrasting perspectives. The interviewer was also external, unknown to them, and created an environment in which they could offer their honest opinions about the training. Second, only one person was directly involved in the coding and analysis of the data, and although measures were in place such that other team members reviewed the data to provide checks on the data, no formal analysis of the reliability of the coding was conducted. One potential issue to keep in mind is that the trainees were overwhelmingly women-identified; thus, the perspectives presented here could underrepresent the perspectives and experiences of men. However, the original sample also was skewed in terms of gender, so it likely is representative of the original sample. It is unclear why the training was more appealing to women than men, or it may be that the recruitment sites were more frequented by women. Future trainings may consider recruiting and/or oversampling men to ensure their perspectives are also represented. Finally, it is worth noting that the trainees expressed confidence in their knowledge of research before the training. This might mean that the people interested in/drawn to participate in the study, and/or those excluded from the study after the reading proficiency screening are not fully representative of “average” community members. Some level of interest and a basic level of confidence in or understanding of research may be necessary for successful trainings.

### Implications for community-engaged research

While efforts are increasingly being made to include community perspectives in research dissemination and recent work attempts to provide helpful guides to assist new or non-scientifically trained reviewers [30, 31], our findings indicate that having strong guidance and support in reviews is necessary. Support, advice, and resources have been noted as important in guidelines on how patients may become involved with research [30]. An important part of our trainees’ experience involved being supported by the training team and interacting with other reviewers/trainees. Our findings should transfer well to programs conducting similar training initiatives with community members. The training is replicable and integrating community perspective into research dissemination could be more effectively implemented and evaluated if such training programs were used more widely. Integrating community perspectives into published research, especially journals publishing community-engaged research, is both important and necessary to ensure the utility of research efforts and justify the expenditure of research funds based on perceived community benefit.

The project detailed here was included as part of a dissemination grant funded by NIH. While some funders encourage community engagement in research activities [32], others specifically require researchers to establish collaborations and/or partnerships with community members (such as the U54 mechanism) in conducting research and/or share research findings with study participants [33, 34]. However, funders should more widely consider requiring the integration of training community reviewers specifically into funding conditions to support embedding community reviewers as a routine part of peer reviews during dissemination. This would allow the findings of this research to be directly implemented, expand this currently limited practice, and maximize the usefulness of research findings.

## Conclusion

Training community members as journal reviewers is innovative and offers the potential to bring unique voices to the traditionally academically focused realm of journal article publishing. Trainees benefit from the training, are well-prepared to review if given a structured curriculum, appropriate tools, strategies, and are supported by a responsive institutional environment. Training programs should emphasize the learning opportunity, include authors’, editors’, and funders’ perspectives, use time carefully, employ effective trainers, and complement didactic training with interactive group work. Employing such strategies can increase the likelihood of community members’ voices being included in research dissemination and increase medical journal articles’ community relevance.

## Data Availability

Data are available on request.
